# Non-homologous end-joining-deficient filamentous fungal strains mitigate the impact of off-target mutations during the application of CRISPR/Cas9

**DOI:** 10.1128/mbio.00668-23

**Published:** 2023-07-24

**Authors:** Sandra Garrigues, Mao Peng, Roland S. Kun, Ronald P. de Vries

**Affiliations:** 1 Fungal Physiology, Westerdijk Fungal Biodiversity Institute and Fungal Molecular Physiology, Utrecht University, Utrecht, the Netherlands; Oswaldo Cruz Foundation, Curitiba, Brazil

**Keywords:** *Aspergillus niger*, biosafety, CRISPR-Cas9, risk assessment, Δ*kusA*

## Abstract

CRISPR/Cas9 genome editing technology has been implemented in almost all living organisms. Its editing precision appears to be very high and therefore could represent a big change from conventional genetic engineering approaches. However, guide RNA binding to nucleotides similar to the target site could result in undesired off-target mutations. Despite this, evaluating whether mutations occur is rarely performed in genome editing studies. In this study, we generated CRISPR/Cas9-derived filamentous fungal strains and analyzed them for the occurrence of mutations, and to which extent genome stability affects their occurrence. As a test case, we deleted the (hemi-)cellulolytic regulator-encoding gene *xlnR* in two *Aspergillus niger* strains: a wild type (WT) and a non-homologous end-joining (NHEJ)-deficient strain Δ*kusA*. Initial phenotypic analysis suggested a much higher prevalence of mutations in the WT compared to NHEJ-deficient strains, which was confirmed and quantified by whole-genome sequencing analysis. Our results clearly demonstrate that CRISPR/Cas9 applied to an NHEJ-deficient strain is an efficient strategy to avoid unwanted mutations.

**IMPORTANCE:**

Filamentous fungi are commonly used biofactories for the production of industrially relevant proteins and metabolites. Often, fungal biofactories undergo genetic development (genetic engineering, genome editing, etc.) aimed at improving production yields. In this context, CRISPR/Cas9 has gained much attention as a genome editing strategy due to its simplicity, versatility, and precision. However, despite the high level of accuracy reported for CRISPR/Cas9, in some cases unintentional cleavages in non-targeted loci—known as off-target mutations—could arise. While biosafety should be a central feature of emerging biotechnologies to minimize unintended consequences, few studies quantitatively evaluate the risk of off-target mutations. This study demonstrates that the use of non-homologous end-joining-deficient fungal strains drastically reduces the number of unintended genomic mutations, ensuring that CRISPR/Cas9 can be safely applied for strain development.

## INTRODUCTION

Filamentous fungi are widely used as cell factories for the production of industrially relevant compounds, such as organic acids, proteins, enzymes, and secondary metabolites (1, 2). One fundamental aspect for the future of fungal biotechnology is the improvement of production strains by precise genetic engineering. The simplicity of the CRISPR/Cas (clustered regularly interspaced short palindromic repeats and CRISPR-associated proteins) systems has been key to widespread implementation of this genome-editing approach in almost all living organisms, including filamentous fungi. The precision that is possible by CRISPR/Cas9 systems represents a big change from conventional genetic engineering approaches, which largely rely on random chemical- or radiation-induced mutagenesis, or untargeted insertions of genes into the genomes through genetic engineering.

CRISPR/Cas9 genome-editing technology is based on the ability of the Cas9 nuclease to cut a specific DNA sequence, generating double-strand breaks (DSBs). Its specificity relies on a 20-bp guide RNA (gRNA) sequence that is designed to target a genetic sequence through the interaction with the protospacer adjacent motive (PAM) “NGG.”

After the DNA break takes place, DSBs are detected as potential damage and have to be repaired for the survival of the species. In fungi, two DNA repair mechanisms exist: the error-prone non-homologous end-joining (NHEJ) and homology-directed repair (HDR). With NHEJ, DNA breaks are re-ligated by the Ku70 and Ku80 heterodimeric protein complex, frequently leading to imprecise DNA repair and loss of genetic functionality (3). In contrast, HDR occurs in the presence of a homologous duplex DNA template (referred to as donor DNA, dDNA) via homologous recombination (HR) (4), which can be used to precisely introduce or remove the DNA sequence of interest in the target organism.

Biosafety should be a central feature of emerging biotechnologies to minimize unintended consequences. Genetic mutations can randomly appear through normal cell division, especially when species are cultured for many generations in the laboratory (5). Additionally, random mutations may also be caused by the mutagenic potential of the transformation process. In fact, it has been reported that transformation enhances the appearance of genetic variations, including chromosomal rearrangements, in different organisms such as bacteria (6), plants (7), and fungi (8, 9). Therefore, ensuring a safe application of CRISPR technologies in the target organisms is a key factor to consider. Despite the high level of accuracy reported for CRISPR/Cas9, annealing of the gRNA to sequences with similarity to the target sequence leads to unspecific DNA recognition and could result in unintentional cleavages in non-targeted loci, known as off-target mutations, thus restricting its application (10). It has been reported that gRNA can tolerate sequence mismatches that still allow Cas9 DSBs generation (11). Basically, the “seed region” (the first 8–12 PAM proximal nucleotides) of the gRNA is vital for recognition, and the potential of off-targeting increases when the mismatches locate further away from the PAM sequence (11). There are some reports of unintended Cas9-induced off-target effects in different organisms, including plants (12, 13), animal, and human cell lines (10, 14), and to an even lesser extent in filamentous fungi (15, 16) and yeast (17). Searching for off-target mutations is far from routine during the experimental procedures in most studies, sometimes leading to unexpected phenotypes misattributed to the originally intended mutation. In filamentous fungi, the off-target analysis has only been partially exploited in *Ustilago maydis* (15), *Aspergillus fumigatus* (9), *Magnaporthe oryzae* (4), and *Trichoderma reesei* (16). However, in all these cases, the number of mutants analyzed is too small to draw robust conclusions, and the off-target effects were analyzed only in wild-type (WT) strains. In this study, we analyzed a large set of CRISPR/Cas9-derived fungal mutant strains to determine whether CRISPR/Cas9 itself induces off-target mutations, and to which extent genome stability plays a role in the accumulation of off-target or random mutations. As a test case, we deleted the (hemi-)cellulolytic regulator-encoding gene *xlnR* (18) in two strains of the industrial workhorse *Aspergillus niger*: a WT strain and an NHEJ-deficient strain in which the *kusA* gene was deleted. The *A. niger kusA* gene encodes the ortholog of the Ku70 protein in other eukaryotes, and its deletion has resulted in an improved homologous integration efficiency of more than 80% compared to the 7% reported in *A. niger* WT with no negative effect on growth and biological cycle of the fungus (19). XlnR is a transcription factor involved in the process of (hemi-)cellulose utilization in ascomycete fungi, and was first identified in *A. niger* (18), being one of the best characterized regulators in this species. Additionally, XlnR together with AraR controls the pentose catabolic pathway, which is required for the utilization of D-xylose and L-arabinose (20). Additionally, nine more regulator-encoding genes (*araR*, *gaaR*, *rhaR*, *galX*, *amyR*, *inuR*, *clrB*, and *creA*) (21) were stepwise deleted in the Δ*kusA* strain. Finally, the effect of the transformation process and the presence of Cas9 in the accumulation of genetic mutations, as well as the off-target mutations caused by CRISPR/Cas9 in both genetic backgrounds or in the Δ*kusA* strain upon consecutive application of CRISPR/Cas9, were assessed.

## RESULTS AND DISCUSSION

### Phenotypic characterization of CRISPR/Cas9-derived transformants points to an increase in the number of mutations in *A. niger* wild type

Key safety and technical considerations for CRISPR/Cas9 application include genomic specificity and editing efficiency (22). In this study, we aimed to evaluate whether the application of a transient CRISPR/Cas9 system induces off-target mutations in the filamentous ascomycete fungus *A. niger*, and how different genomic backgrounds could affect the accumulation of these and other random mutations.

We used CRISPR/Cas9 plasmids harboring both *cas9* and gRNA to effectively target *xlnR*, and dDNA was also included to allow DNA repair by HR. Deletion mutants of *xlnR* gene have been shown to grow poorly on xylan as the sole carbon source (23, 24). Therefore, this makes *xlnR* a good candidate for its disruption using CRISPR/Cas9, as it facilitates transformant selection by phenotypic characterization. Additionally, for control experiments, *A. niger* strains resulting from transformations using empty plasmids (with no *cas9*)*,* and plasmids with *cas9* but no gRNA were included.

**Fig 1:**
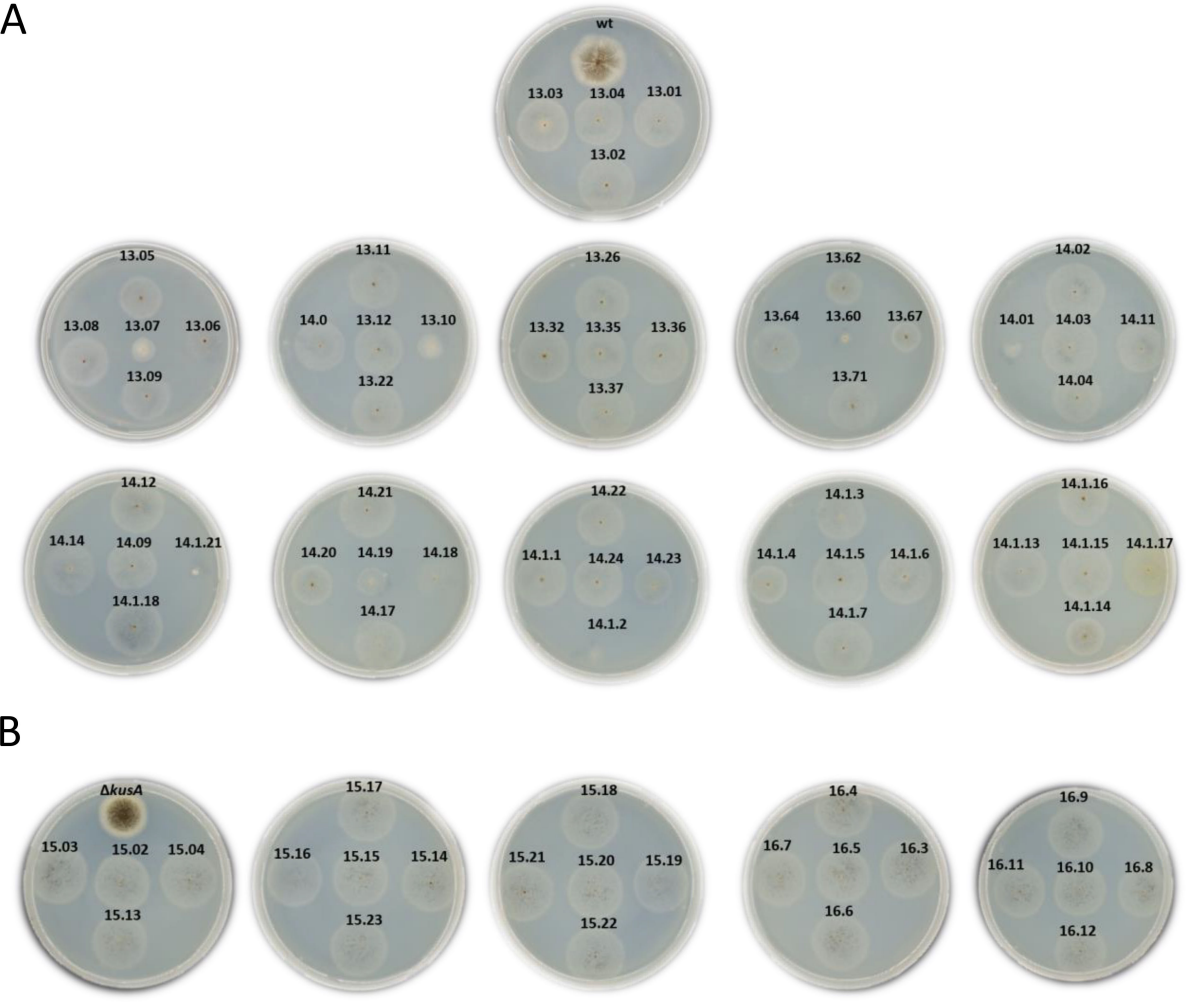
Phenotypic characterization of *A. niger* Δ*xlnR* strains in wild-type (**A**) and Δ*kusA* (**B**) genetic backgrounds. Strains were grown on 1% xylan-containing minimal medium plates for 4 days at 30°C.

All the 78 mutants generated after transformation with CRISPR/Cas9 plasmids containing *cas9* and gRNA were analyzed on xylan-containing plates (Fig. 1) and confirmed by PCR (Fig. S1). From these 78 mutants, 54 Δ*xlnR* mutants were obtained in *A. niger* WT genetic background (Fig. 1A), while 24 were obtained in the NHEJ-deficient strains Δ*kusA* (Fig. 1B). All of them showed reduced growth on xylan plates, confirming the disruption of the targeted gene and 100% on-target activity of the designed gRNA. However, 13 WT-derived *A. niger* Δ*xlnR* strains (ID 13.06, 13.07, 13.10, 13.60, 13.67, 14.01, 14.18, 14.19, 14.23, 14.1.2, 14.1.4, 14.1.14, and 14.1.21) showed unexpected growth phenotypes after 4 days of growth (Fig. 1A). In contrast, none of the Δ*xlnR* mutants generated in the NHEJ-deficient strains showed unexpected growth phenotypes under the same culture conditions (Fig. 1B). Taken together, these results may indicate that the aberrant phenotypes observed in the 24% of the WT-derived Δ*xlnR* strains obtained are as consequence of the application of CRISPR/Cas9. Also, these results preliminary suggested a higher genetic instability of the WT genome in terms of mutation accumulation, in which both NHEJ and HDR DNA repair mechanisms take place, compared to the NHEJ-deficient strains, in which only HDR is possible. To further confirm these hypotheses, a selection of control and transformant strains from both genetic backgrounds were selected for whole-genome sequencing (WGS) and analyzed for the presence of genetic variants (see Fig. S1 and sections below).

### The genetic variations induced by the transformation process are mitigated in NHEJ-deficient *A. niger* strains

**Fig 2:**
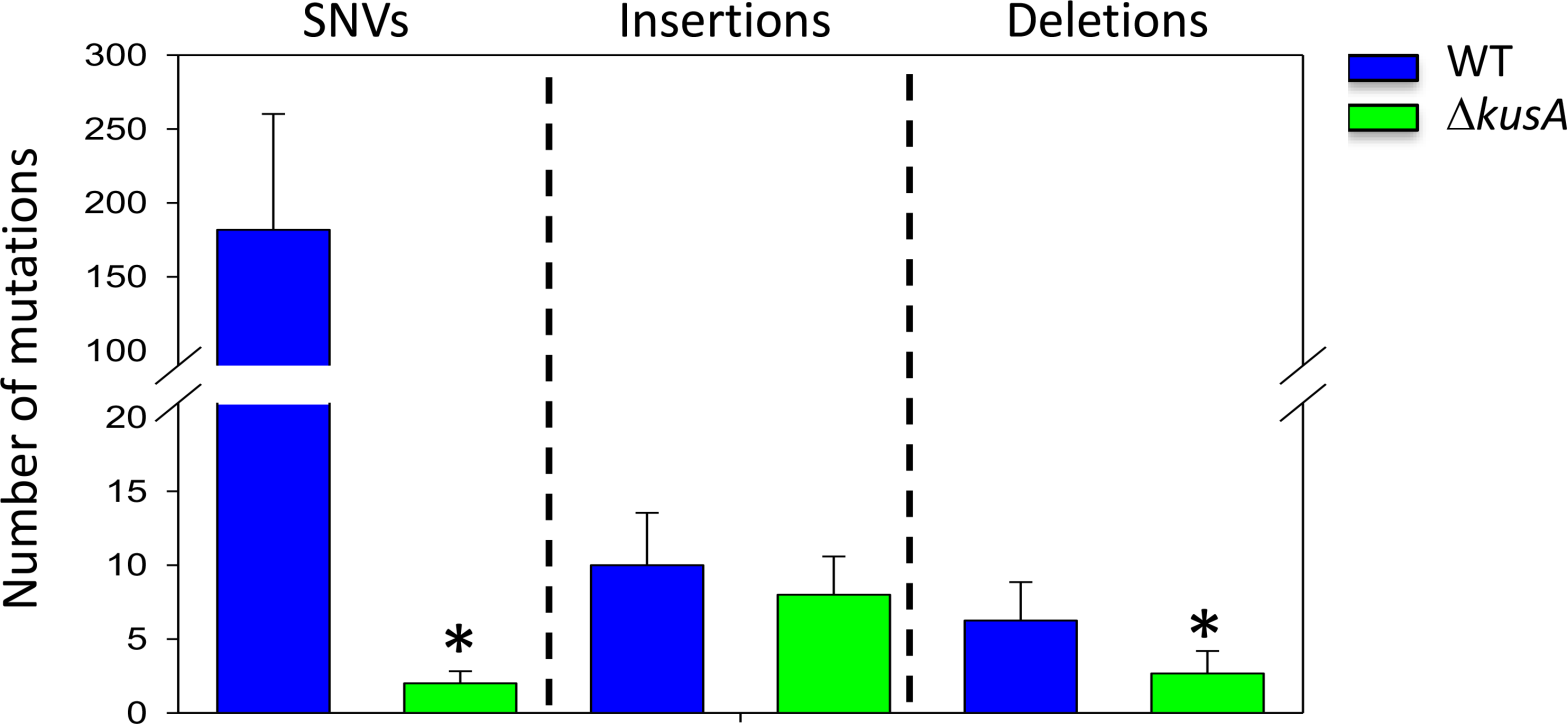
Average number of random mutations caused by the transformation process. Average number of SNVs, insertions and deletions of the wild-type (WT, blue bars), and the Δ*kusA* strain (green bars) that were transformed with the ANEp8-*pyrG* plasmid in which *cas9* is not present. Error bars represent the standard deviation (SD) of the replicate samples. Asterisks represent statistical significance for each of the different mutations (*t* test, *P* < 0.05). Note the break at *Y*-axis.

In this study, we transformed both *A. niger* WT and Δ*kusA* backgrounds with the empty ANEp8-*pyrG* plasmid, which did not contain *cas9*, and analyzed the resulting strains by WGS to determine the frequency of genetic mutations that may occur due to the transformation process. This also provides a baseline to compare the possible impact of CRISPR/Cas9 on off-target/random mutations. Comparative bioinformatics analyses revealed that multiple genomic mutations were present in both genetic backgrounds, accumulating single-nucleotide variants (SNVs), insertions, and deletions after the protoplast-mediated transformation process (Fig. 2). However, the average number of genetic variations detected in the Δ*kusA* strains (2 SNVs, 8 insertions, and 2 deletions) was generally lower compared to the WT (181 SNVs, 10 insertions, and 6 deletions) (Table S1). In this context, there was a statistically significant difference in the average number of detected SNVs and deletions between the WT and the Δ*kusA* SNVs (*P* = 0.001) and deletions (*P* = 0.034) between the WT and the Δ*kusA* strain after transformation (Table S3). These results confirm the occurrence of genetic mutations during the transformation process used in this study, which is a polyethylene glycol (PEG)-mediated transformation procedure commonly used by the scientific community nowadays not only for *Aspergillus* species (9, 25 - 27), but also for *Penicillium* (28 - 32), *Talaromyces* (33), and more phylogenetically distant fungal species such as *Trichoderma* (34), *Neurospora* (35), *Stagonospora* (36), and *Dichomitus* (37), for instance. This standard transformation protocol might induce cellular stress as it requires the enzymatic digestion of the cell wall for protoplast release, followed by recovery on an osmotically stabilized agar medium (9). Additionally, these results indicate that by disrupting the NHEJ repair mechanism, the genomic integration of mutations is prevented, severely reduced, and/or cannot survive in the resulting transformants. This supports a previous suggestion (38) that the Δ*kusA* genetic background is a more stable genotype, and therefore more suitable for further transformation procedures in terms of mutation accumulation.

Finally, these results can provide an explanation for the percentage of WT Δ*xlnR* strains with unexpected morphologies detected after phenotypic analyses (see previous section), which can be attributed to the higher mutation accumulation rate in the WT.

### The presence of Cas9 does not increase occurrence of mutations in *A. niger*

Apart from mutation that occurs due to the transformation process, we wanted to assess if the presence of Cas9 would increase the occurrence of genetic mutations, as increasing evidence has shown that high levels of Cas9 protein may have a toxic effect to host cells. In this sense, Cas9 endonuclease has been reported to exert toxicity against several organisms such as *Saccharomyces cerevisiae* (39), *Cryptococcus neoformans* (40), *M. oryzae* (4), and *Fusarium venenatum* (41). Additionally, some authors demonstrated that increasing Cas9 concentrations in the presence of functional gRNAs did not increase the occurrence of genomic mutations in *A. fumigatus* (9). However, there are no studies in which the genotoxicity of Cas9 without the presence of functional gRNAs that drive Cas9 endonuclease activity is reported to date.

**Fig 3:**
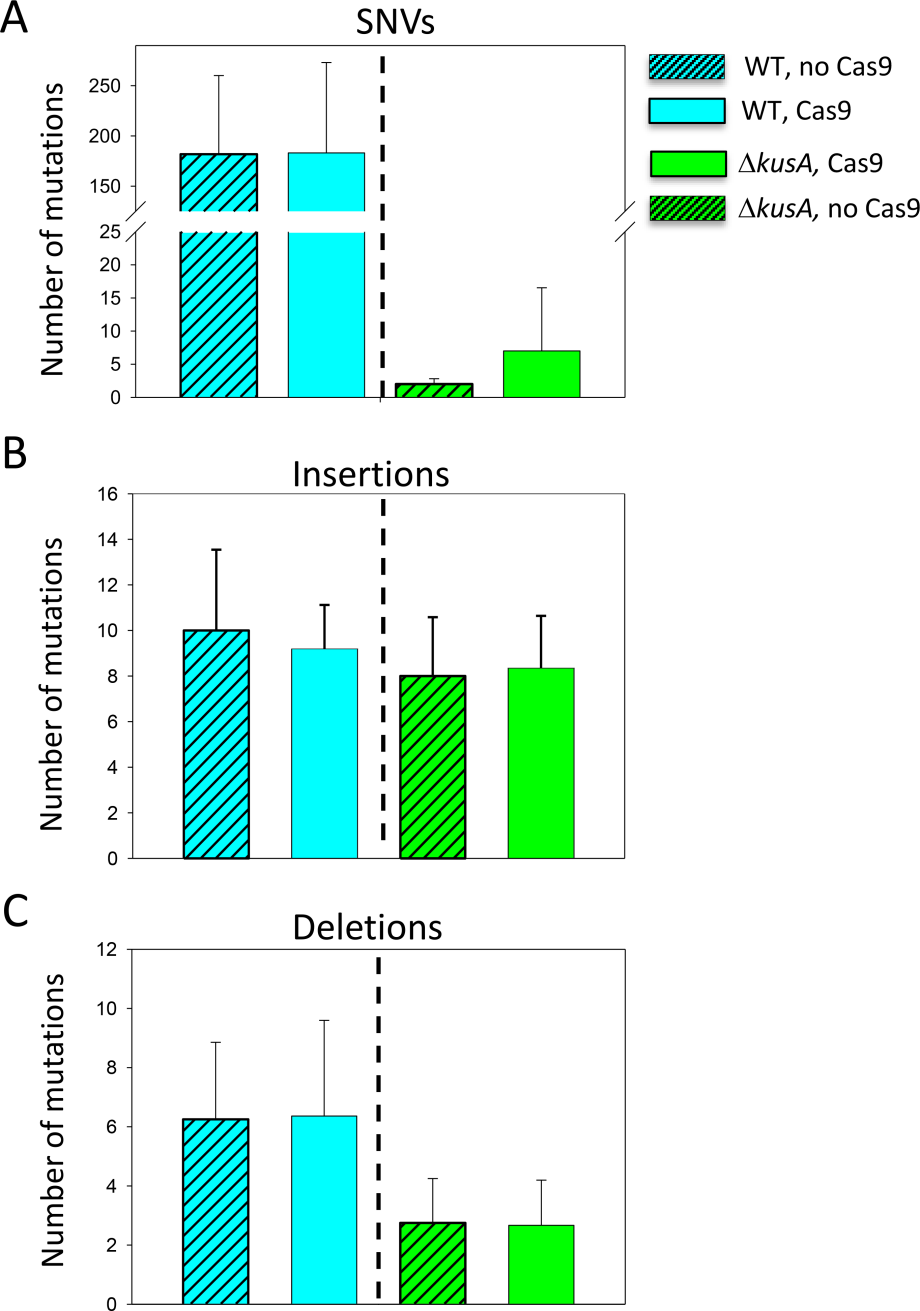
Assessment of the number of random mutations caused by the presence of Cas9. Average number of SNVs (**A**), insertions (**B**), and deletions (**C**) in the wild-type (WT, blue bars) and Δ*kusA* strains (green bars) detected after transformation in the absence (patterned bars) or presence (solid bars) of Cas9 endonuclease. Error bars represent the standard deviation (SD) of the replicate samples. No difference in significance was found in any analysis (*P* < 0.05). Note the break in *Y*-axis of panel **A**.

To determine if the presence of Cas9 (without any gRNA) in the cells increases the occurrence of genetic mutations, we compared and statistically analyzed the genetic variation identified after transformation in the presence and absence of Cas9 between the WT and Δ*kusA* strains. As shown in Fig. 3, there was no significant increase in the number of SNVs, insertions, and deletions when the ANEp8-Cas9-*pyrG* plasmid was transformed into the cells compared to the number of variations shown after transformation with an empty plasmid in any of the genetic backgrounds under study (*P* > 0.05) (Table S2). Thus, we show for the first time that the presence of Cas9 does not aggravate the occurrence of mutations in the absence of gRNAs. Additionally, the results confirmed that *A. niger* Δ*kusA* genetic background accumulates fewer genetic mutations, particularly SNVs (Fig. 3A, green bars) (Table S1), further demonstrating the higher genomic stability of the NHEJ-deficient strains compared to the WT in terms of mutation accumulation.

### NHEJ-deficient strains mitigate the impact of unintended mutations after the application of CRISPR/Cas9

To date, the putative occurrence of off-target mutations caused by CRISPR/Cas9 in the industrial workhorse *A. niger* had not been addressed. In addition, it has not been addressed so far whether the DNA repair mechanism used to repair DSBs or the use of NHEJ-deficient fungal strain would affect the occurrence of off-target mutations after CRISPR/Cas9 genome editing. In this study, we wanted to address these issues by comparing the number of genetic variants obtained in: (i) *A. niger* WT genetic background that repaired DSBs at the *xlnR* locus by NHEJ compared to HDR; (ii) the WT and Δ*kusA* genetic backgrounds each of them transformed with *cas9-*containing plasmid with no gRNA compared to that transformed with a functional CRISPR/Cas9 system; and (iii) CRISPR/Cas9-derived Δ*xlnR* strains in the WT compared to the Δ*kusA* genetic background.

**Fig 4:**
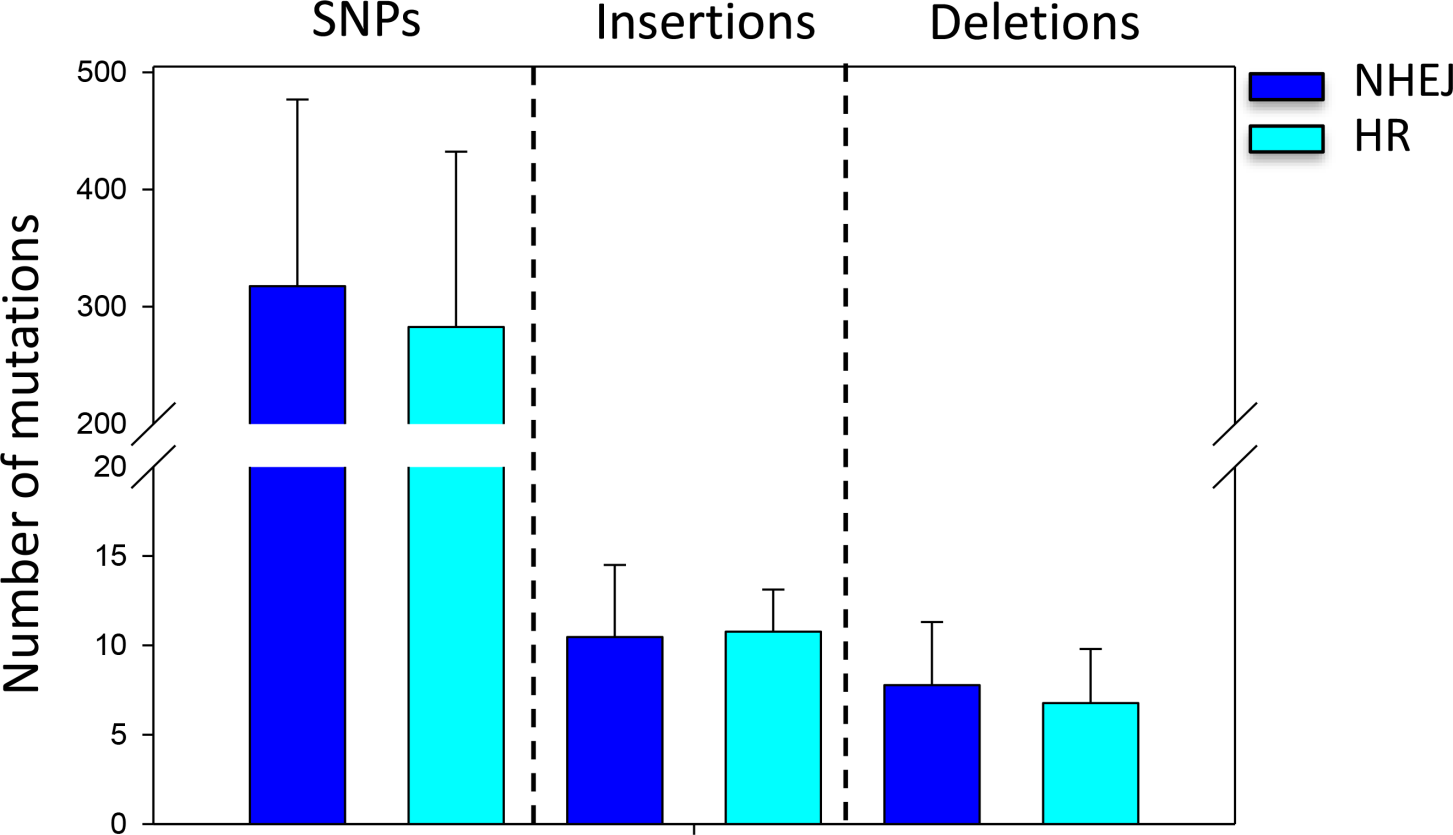
Mutations arising in the wild type (WT) after *xlnR* disruption with CRISPR/Cas9 depending on the DNA repair mechanism. Average number of SNPs, insertions, and deletions in the WT Δ*xlnR* strains that underwent NHEJ (dark blue bars) or HR (light blue bars). Error bars represent the standard deviation (SD) of the replicate samples. No difference in significance was found in any analysis (*P* < 0.05). Note the break in *Y*-axis.

First, in order to analyze whether the DNA repair mechanisms influenced the number of genetic variants accumulated in the WT after a single CRISPR/Cas9 event, strains were transformed with the ANEp8-Cas9-*pyrG* plasmid that contained a *xlnR-*gRNA cassette together with a dDNA. In this situation, some strains repaired the DSBs via NHEJ, whereas others did this by HDR using the dDNA. NHEJ commonly resulted in short deletions and insertions, while the HDR resulted in full deletions (see example in Fig. S2). Results showed that there was no significant difference in the number of SNVs, insertions, or deletions (*P* > 0.05) in the WT strain regardless of the repair pathway (Fig. 4; Table S2). These results confirmed that neither the DNA repair mechanism, nor the presence of dDNA influenced the occurrence of mutations in the WT genetic background. Since there was no significant difference, these two categories were combined into a single category for subsequent analyses.

**Fig 5:**
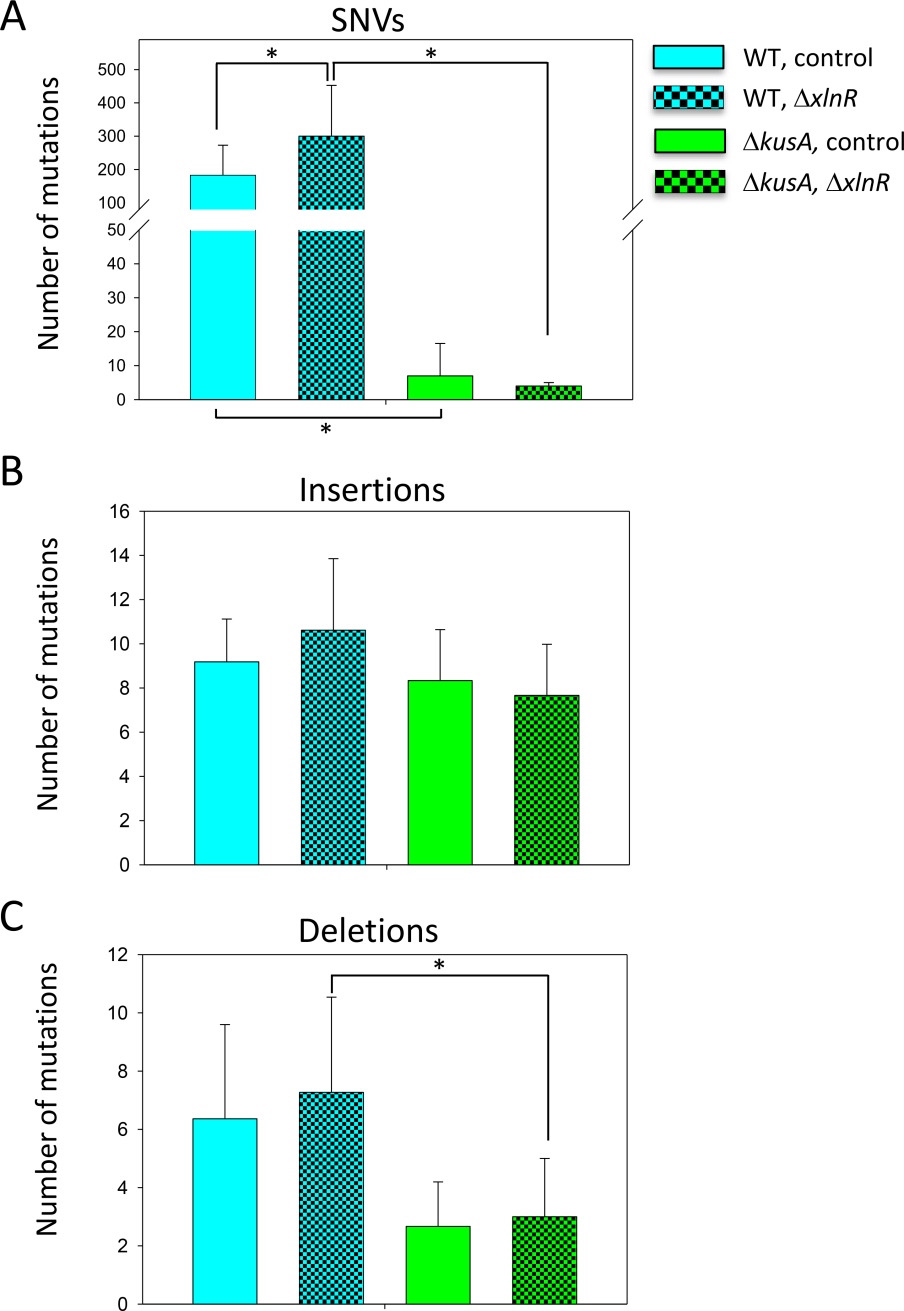
Comparison of the number of mutations in *xlnR* deletion strains in the two different genetic backgrounds. Average number of SNVs (**A**), insertions (**B**), and deletions (**C**) in the wild-type strains (WT, blue bars) and in Δ*kusA* (green bars) which received the cas9-plasmid ANEp8-pyrG_*cas9* without any functional guide (solid bars, control) and the strains that were transformed with the ANEp8-*cas9-pyrG-xlnR* plasmid (patterned bars). Error bars represent the standard deviation (SD) of the replicate samples. Difference in significance is represented by *P < 0.05. Note the break in *Y*-axis of panel **A**.

In parallel, we compared the two different background strains in which the *xlnR* gene was disrupted by CRISPR/Cas9 with their corresponding control strains that were transformed with *cas9*-containing plasmid but no gRNA. This way, we can determine if there is a significant increase in the number of mutations after the application of a functional CRISPR/Cas9 system within the cells. As shown in Fig. 5, there was a significant increase in the number of SNVs in the WT genetic background after the application of a functional CRISPR/Cas9 genome-editing event compared to the control (*P* = 0.023), with a 0.6-fold increase in this case (Table S1), while the number of insertions and deletions is more or less maintained and not significantly different in this genotype (Fig. 5, blue bars) (Table S2). In contrast, the number of SNVs, insertions, and deletions did not significantly change after the application of CRISPR/Cas9 in the NHEJ-deficient strains (Fig. 5, green bars) (Tables S1 and S2). Besides, there is statistical difference in the average number of SNVs and deletions between the WT Δ*xlnR* and Δ*kusA* Δ*xlnR* strains (*P* = 0.002 and *P* = 0.037, respectively), with a lower number of these mutations in the Δ*kusA* background strains (Fig. 5; Table S1). These results suggest that in the *A. niger* WT genetic background, there is a higher chance of genetic variant accumulation after the application of CRISPR/Cas9, which is mitigated in Δ*kusA*. Moreover, the number of mutations accumulated in Δ*kusA* is generally much lower than those in the WT, demonstrating that Δ*kusA* is a safer option when applying CRISPR/Cas9 in terms of mutation accumulation rate.

**Fig 6:**
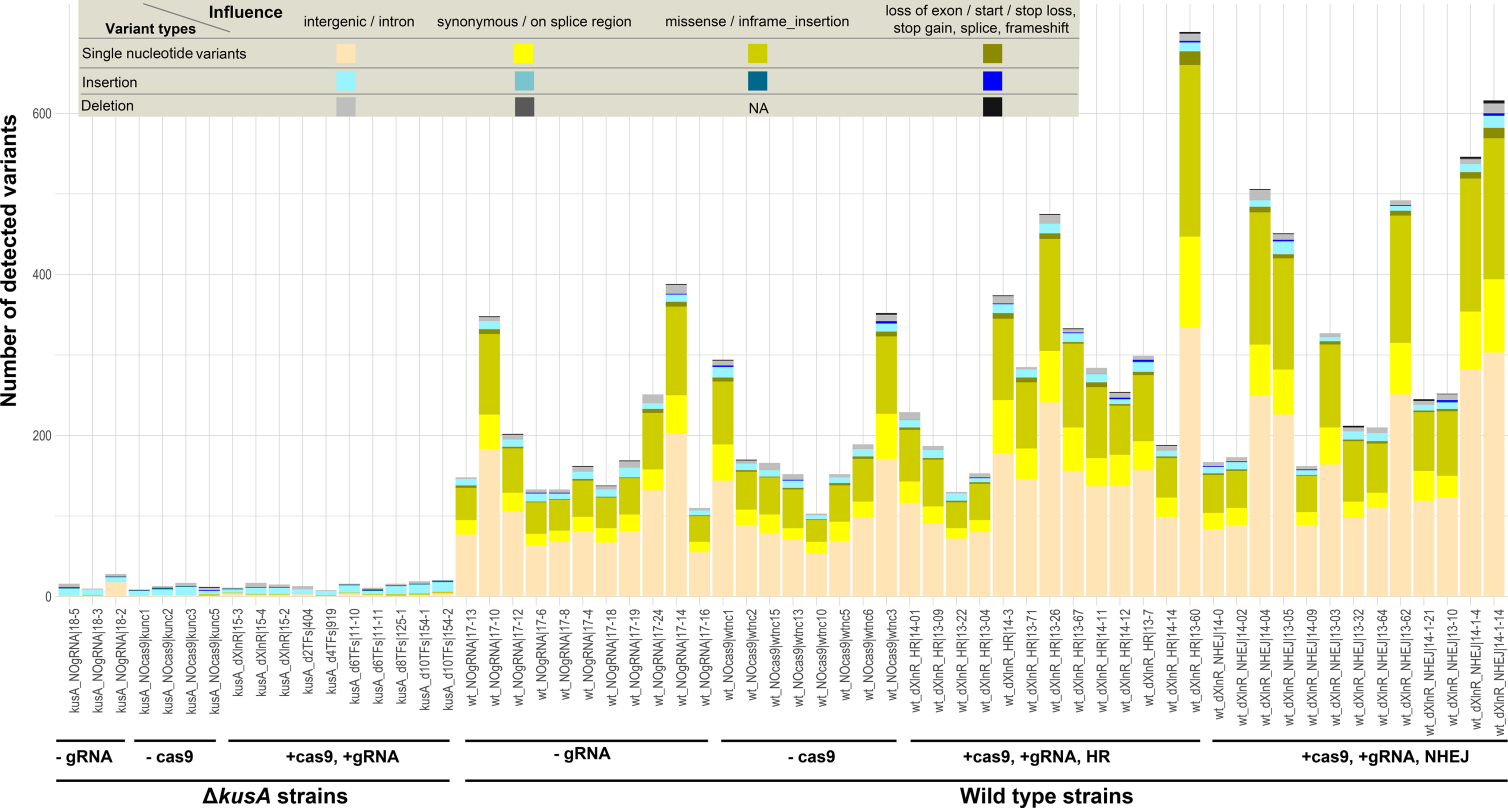
Distribution of the different types of genomic variants detected in each of the sequenced *A. niger* strains.

Having demonstrated the higher occurrence of mutation accumulation in the WT strain after the transformation process and the CRISPR/Cas9 application, we aimed to address the biological influence of the different variant types according to their location in the genome (Fig. 6). For the WT-derived strains, the majority of genetic variants detected (88–95%) are SNVs (Fig. 6, in green; Table S1). Whereas approximately half of the SNVs occurred in the intergenic or intron regions, the other major detected SNVs were related to splicing and protein-coding regions, and a small ratio of the detected SNVs was related to changes of start or stop codons or frameshift. Overall, many of these mutations may cause moderate effects on the function of the corresponding proteins, thus, resulting in unexpected morphologies that in the practice could be misattributed to the originally intended mutations. Noteworthy, we have detected a small number of insertion and deletion variants, which mainly occur in non-coding regions. The overall numbers and distribution of variants are consistent with a previous study performed in *A. fumigatus* (9). In contrast, we observed a much lower ratio of SNVs linked to non-coding regions in *A. niger* (~50%) than in *A. fumigatus* (~96%), which can be attributed to differences between species and/or analysis methods. In contrast, in the Δ*kusA* strains, only a limited number of genomic variants were detected. In these strains, SNVs only contributed to 10–30% of total variants (except for one sample with a ratio of 62%), with insertions being the predominant variants (Fig. 6, in blue; Table S1). Remarkably, most of the mutations took place in intergenic/intronic regions. Therefore, there is a small chance of affecting gene functionality.

To further examine the genetic variants identified in both genetic backgrounds, we compared them with the predicted CRISPR/Cas off-targets using Cas-OFFinder. None of the variants were identified as putative Cas9 off-target by the software. However, we cannot discard the possibility that some of these mutations would have been caused by CRISPR/Cas9, especially in the WT, in which the mutation rate is much higher. In any case, using NHEJ-deficient strains mitigates the problem of mutation occurrence observed in the WT.

Editing of multiple loci is often required to either introduce multiple heterologous genes or fine-tune metabolic networks of microbial cell factories. The establishment of recyclable, marker-free CRISPR-Cas9 tools as the one used in this study (38) brought powerful genome engineering capabilities, considerably saving time and resources in strain construction programs (42). However, it has not been studied yet whether consecutive CRISPR/Cas9 transformations induced a higher accumulation of mutations compared to single transformations. For this, we stepwise obtained a 10-deletion mutant in the 10 studied transcription factors involved in plant biomass degradation (∆*araR*∆*gaaR*∆*rhaR*∆*galX*∆*xlnR*∆*clrB*∆*amyR*∆*inuR*∆*clrA*∆*creA*) (21) after five consecutive transformations using CRISPR/Cas9 in the NHEJ-deficient strain, in which we already saw a significant decrease in the accumulation of unintended mutations. Finally, the 10-deletion strain together with the intermediate 4-, 6-, and 8-deletion strains (see Materials and Methods) was analyzed by WGS (Fig. S3). Comparative analysis of the multi-deletion strains revealed again that only a small set of genetic variants accumulated in *A. niger* Δ*kusA* strains (Fig. S3A). Besides, all the 10 targeted transcription factors were precisely deleted, and only 23 extra genetic variants were accumulated after five rounds of consecutive CRISPR/Cas9 gene editing (Fig. S3B). All these data strongly support that NHEJ-deficient strains significantly mitigate the impact of the occurrence of unintended mutations during the application of CRISPR/Cas9.

Several studies have reported that numerous undesired mutations linked to CRISPR/Cas9 in different organisms (13, 43 - 45). Nevertheless, the assessment of CRISPR/Cas9-derived off-target mutations in filamentous fungi has been poorly exploited to date. In *A. fumigatus* WT, after WGS of six independent mutants, the CRISPR/Cas9 system was reported as a highly efficient and reliable method of gene targeting with no associated off-target mutations (9). In another study, after sequencing two independent CRISPR/Cas9-derived *M. oryzae* WT mutants, authors concluded that no off-target CRISPR-generated mutations occurred (4). In *T. reesei*, a constitutively active CRISPR/Cas9 system was reported to generate off-target mutations close to the sgRNA target site in the *ura5* gene in seven independent mutants, although no WGS of these strains was performed (16). Finally, WGS of two independent *bw2* knock-out strains revealed no obvious off-target effects in *U. maydis* (15). However, in all these studies, the number of mutants analyzed was very small to draw robust conclusions and only WT strains were taken into account. In this context, our study is the first one showing the occurrence of mutations in *A. niger* and the protective effect of the Δ*kusA* strains in the accumulation of such mutations in a large and representative set of CRISPR/Cas9-derived mutants.

Several research groups have put such an effort in developing technical solutions to address CRISPR/Cas9 safety concerns. These include reducing the levels of active Cas9 (46), reducing Cas9 lifetime (47, 48), shortening the gRNA sequence at the 5′-end region (49), or generating high fidelity Cas9 nucleases such as *Sp*Cas9-FokI, *Sp*Cas9-HF1, and *eSp*-Cas9, among others [reviewed in reference (50)]. In addition, optimum gRNA design is crucial to maintain editing specificity.

Bioinformatic tools are now available to search for potential Cas9 off-target sites, including Cas-Designer (51) and Cas-OFFinder (52), which we used in our study. Also, studies have been published on ways to minimize off-target mutations in CRISPR/Ca9 systems. For example, CRISPR Guide RNA Assisted Reduction of Damage (CRISPR GUARD) has been recently described as a method for protecting off-target sites by co-delivery of short gRNAs directed against off-target loci by competition with the on-target gRNA (53). However, based on our study, we propose that using NHEJ-deficient strains is one of the most efficient strategies to decrease the occurrence of unintended mutations after the application of CRISPR/Cas9 in filamentous fungi, especially for consecutive mutations.

In conclusion, as CRISPR/Cas9 genome-editing technology is widely used in many organisms, including filamentous fungi, the safety of genome-edited strains is a matter of discussion within the scientific community. In this study, we analyzed a large set of CRISPR/Cas9-derived fungal mutant strains to determine whether CRISPR/Cas9 induces off-target mutations in two different *A. niger* genetic backgrounds, the WT and NHEJ-deficient Δ*kusA* strains. Overall, we showed that the transformation process in itself is the most dominant cause of random mutations, but that this can be largely mitigated by using a NHEJ-deficient strain. CRISPR/Cas9 itself is a reliable and safe genome-editing technology when applied in a NHEJ-deficient strain. Thus, we strongly recommend the use of NHEJ-deficient strains during strain engineering due to their much higher genomic stability in terms of mutation accumulation. While we expect that the higher genome stability in NHEJ-deficient strains is a common phenomenon in fungi, similar evaluation of other species as presented here for *A. niger* should be performed to confirm this.

## MATERIALS AND METHODS

### Strains, media, and growth conditions

The ascomycete *A. niger* N593 (*pyrG*^−^, referred to as WT) and *A. niger* CBS138852 (*pyrG^−^ ΔkusA*) strains were used as parentals for transformation. Strains were grown at 30°C in *Aspergillus* minimal medium (MM) or complete medium (CM) (54) supplemented with 1% D-glucose and 1.22 g/mL uridine (Sigma-Aldrich, Zwijndrecht, the Netherlands). Conidia were subsequently harvested, dispersed in sterile Milli-Q H_2_O, and the concentration was determined using a hemocytometer. For phenotypic characterization of the transformants, 250 spores were inoculated on MM plates supplemented with 1% beechwood xylan and 1.22 g/mL uridine (Sigma-Aldrich).

*Escherichia coli* DH5α was used to propagate plasmids, and was grown in Luria-Bertani medium containing 50  µg/mL of ampicillin (Sigma-Aldrich).

### DNA constructs, fungal transformation, and screening of transformants

Plasmid constructions for the generation of *A. niger* deletion strains in the WT and NHEJ-deficient strains were performed as previously described (23). The self-replicative CRISPR/Cas9 plasmid ANEp8-Cas9-*pyrG* was used (AddGene #117169) (38). As control for transformation, the non-*cas9*-containing version of the ANEp8 plasmid (ANEp8-*pyrG,* empty plasmid) was also used. The gRNA sequences to delete *xlnR* (ID: NRRL3_04034), *gaaR* (ID: NRRL3_08195), *araR* (ID: NRRL3_07564), *rhaR* (ID: NRRL3_01496), *galX* (ID: NRRL3_07290), *clrA* (ID: NRRL3_03544), *clrB* (ID: NRRL3_09050), *amyR* (ID: NRRL3_07701), *inuR* (ID: NRRL3_03593), and *creA* (ID: NRRL3_05946) were designed with no predicted oﬀ-targets and the highest on-target activity using Geneious 11.1.4 (https://www.geneious.com) based on the experimentally determined predictive model described by reference (55). The dDNA constructs were obtained by fusion PCR using Phusion High-Fidelity DNA Polymerase (Thermo Fisher Scientific, Bleiswijk, the Netherlands) and include 500–1,000 bp of the 5′ and 3′ flanking regions of the target gene for HR. All primers used were purchased from Integrated DNA Technologies and are listed in Table S3.

*A. niger* protoplast generation and transformation were performed as previously described (27). In brief, 1 µg of either ANEp8-Cas9-*pyrG* or ANEp8-*pyrG* plasmid was transformed into the cells, and 5 µg of the dDNA was co-transformed to allow HR. Different transformation experiments were carried out: (i) both genetic background strains (WT and Δ*kusA*) were transformed with a version of the plasmid that contained the *cas9* gene (ANEp8-Cas9-*pyrG*) without gRNA (ii); both backgrounds were transformed with an empty plasmid that did not contain *cas9* (ANEp8-*pyrG*); and (iii) both genetic backgrounds were transformed with a *cas9*-containing plasmid (ANEp8-Cas9-*pyrG)* and the gRNA cassette for targeting *xlnR* gene with dDNA. Purification of transformant strains was achieved by two consecutive single colony streaking, followed by cultivating the strains on uridine-containing plates to promote the loss of the self-replicating plasmids. Subsequently, transformants were grown on medium containing 5-fluoro-orotic acid in order to screen for colonies which lost the plasmid. For the construction of multi-deletion strains, the *A. niger* Δ*kusA* strain was repeatedly transformed with a *cas9*- and a gRNA cassette-containing plasmid (ANEp8-Cas9-*pyrG*) together with the corresponding dDNA, knocking out two genes in each transformation round, followed by the removal of the self-replicating plasmid. First, the *gaaR* and *araR* genes were simultaneously knocked out. The double deletion strain Δ*gaaR*Δ*araR* was used as background for the deletion of *rhaR* and *galX*, resulting in the quadruple deletion strain Δ*gaaR*Δ*araR*Δ*rhaR*Δ*galX*, which was used for the generation of the Δ*gaaR*Δ*araR*Δ*rhaR*Δ*galX*Δ*xlnR*Δ*clrB* sextuple deletion strain (56). Finally, the octuple Δ*gaaR*Δ*araR*Δ*rhaR*Δ*galX*Δ*xlnR*Δ*clrB*Δ*amyR*Δ*inuR* deletion strain was generated by the deletion of *amyR* and *inuR* in the sextuple deletion strain, while the decuple deletion strain Δ*gaaR*Δ*araR*Δ*rhaR*Δ*galX*Δ*xlnR*Δ*clrB*Δ*amyR*Δ*inuR*Δ*clrA*Δ*creA* was generated by the double deletion of *clrA* and *creA* in the octuple deletion strain (R. S. Kun, S. Garriegues, R. P. de Vries, unpublished data). Genomic DNA from transformants was isolated using Wizard Genomic DNA Purification Kit (Promega, Leiden, the Netherlands), and PCR reactions were performed to analyze the transformant colonies using GoTaq Flexi DNA Polymerase (Promega) following the manufacturer’s instructions.

### Genome sequencing and variant calling

To investigate the possible off-target effects, we sequenced all mutant and reference strains using the Illumina NovaSeq 6000 system at Centre d’expertise et de services Génome Québec (https://cesgq.com/home). The resulting data were 250 bp paired-end reads, which were cleaned up using Fastp v0.23.2 (57) with default parameters.

Variants were called using the Breseq tool with default settings (58, 59). The genome and gene annotations of *A. niger* NRRL3 were used as reference (https://mycocosm.jgi.doe.gov/Aspni_NRRL3_1/Aspni_NRRL3_1.home.html) (60). Variants with quality score lower than 30 were filtered out. Mutations were annotated using SnpEff v5.0 (61). Only the mutations detected that were supported with at least 10 reads and were not present in the initial parental strains (WT or *ΔkusA* strain) were considered for further analysis. To assess whether the detected variants were potentially caused by CRISPR-Cas9, off-targets were predicted using Cas-OFFinder (51) by allowing “three mismatches with one DNA/RNA bulge.” On/off-target mutations were additionally visually confirmed using the Integrative Genomics Viewer (62).

### Statistical analysis

Differences between the samples were analyzed using Student two-tailed *t* test. Statistical analyses were performed using STATGRAPHICS 18 (https://www.statgraphics.com/). Statistical significance was regarded as *P* < 0.05.

## Data Availability

In total, we generated WGS data sets for 65 different strains, including both the reference and CRISPR/Cas9-edited strains. The related sequencing data have been deposited in NCBI Sequence Read Archive (SRA) database under the BioProject accession number PRJNA931827 and SRA accession numbers SRR23345953–SRR23346017.

## SUPPLEMENTAL MATERIAL

Supplemental Figures mbio.00668-23-s0001.pdf Figures S1 to S3 and Tables S1 to S3.

ASM does not own the copyrights to Supplemental Material that may be linked to, or accessed through, an article. The authors have granted ASM a non-exclusive, world-wide license to publish the Supplemental Material files. Please contact the corresponding author directly for reuse.
