# Supplementary material for: Non-homologous end-joining-deficient filamentous fungal strains mitigate the impact of off-target mutations during the application of CRISPR/Cas9
Source: mBio. 2023 Jul 24;14(4):e00668-23. doi: 10.1128/mbio.00668-23 (PMC10470509; doi:10.1128/mbio.00668-23)
Supplement: Supplemental Figures — Figures S1 to S3 and Tables S1 to S3. [file mbio.00668-23-s0001.pdf]

Supplementary Material

Non-homologous End-Joining-deficient filamentous fungal strains mitigate the impact of off-target mutations during the application of CRISPR/Cas9

Sandra Garrigues[a*] [§], Mao Peng[a] [§], Roland S. Kun[a†] and Ronald P. de Vries[a#]

[a]Fungal Physiology, Westerdijk Fungal Biodiversity Institute & Fungal Molecular Physiology, Utrecht University, Uppsalalaan 8, 3584 CT Utrecht, The Netherlands

[*]Present address: Department of Food Biotechnology, Instituto de Agroquímica y Tecnología de Alimentos (IATA), Consejo Superior de Investigaciones Científicas (CSIC), Paterna, Valencia, Spain.

[†]Present address: Leiden University, Institute of Biology Leiden, Microbial Sciences, Fungal Genetics and Biotechnology, Sylviusweg 72, 2333 BE, Leiden, The Netherlands

[§] Authors contributed equally to this work and should be considered first co-authors. Author order is in order of increasing seniority.

[#]Corresponding author: Ronald P. de Vries. E-mail: r.devries@knaw.wi.nl

Running Title: CRISPR/Cas9 risk assessment in fungi

A

B

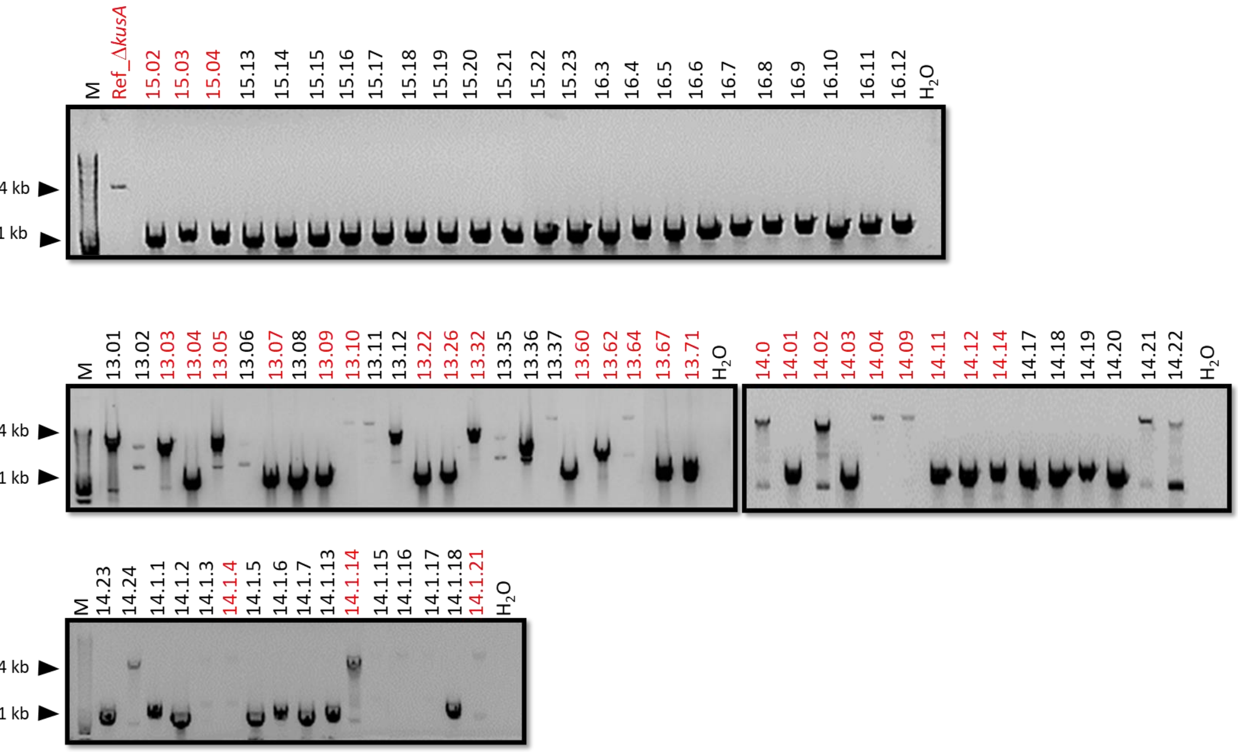

Supp. Fig S1. Molecular characterization of the Δ*xlnR* mutants in the Δ*kusA* (A) and wild-type (B) genetic backgrounds. The 1 kb bands represent clean *xlnR* deletions after HR in the presence of a dDNA. The 4 kb bands represent the presence of *xlnR* gene compatible with NHEJ DNA repair mechanism. Note that all Δ*kusA*-derived strains underwent HR, while wild type-derived strains underwent both HR and NHEJ. PCRs were carried out with oligos An-*xlnR*_5F and An-*xlnR*_3R (Supp. Table S1). Strains for which WGS was successful are represented in red. M: Molecular marker.

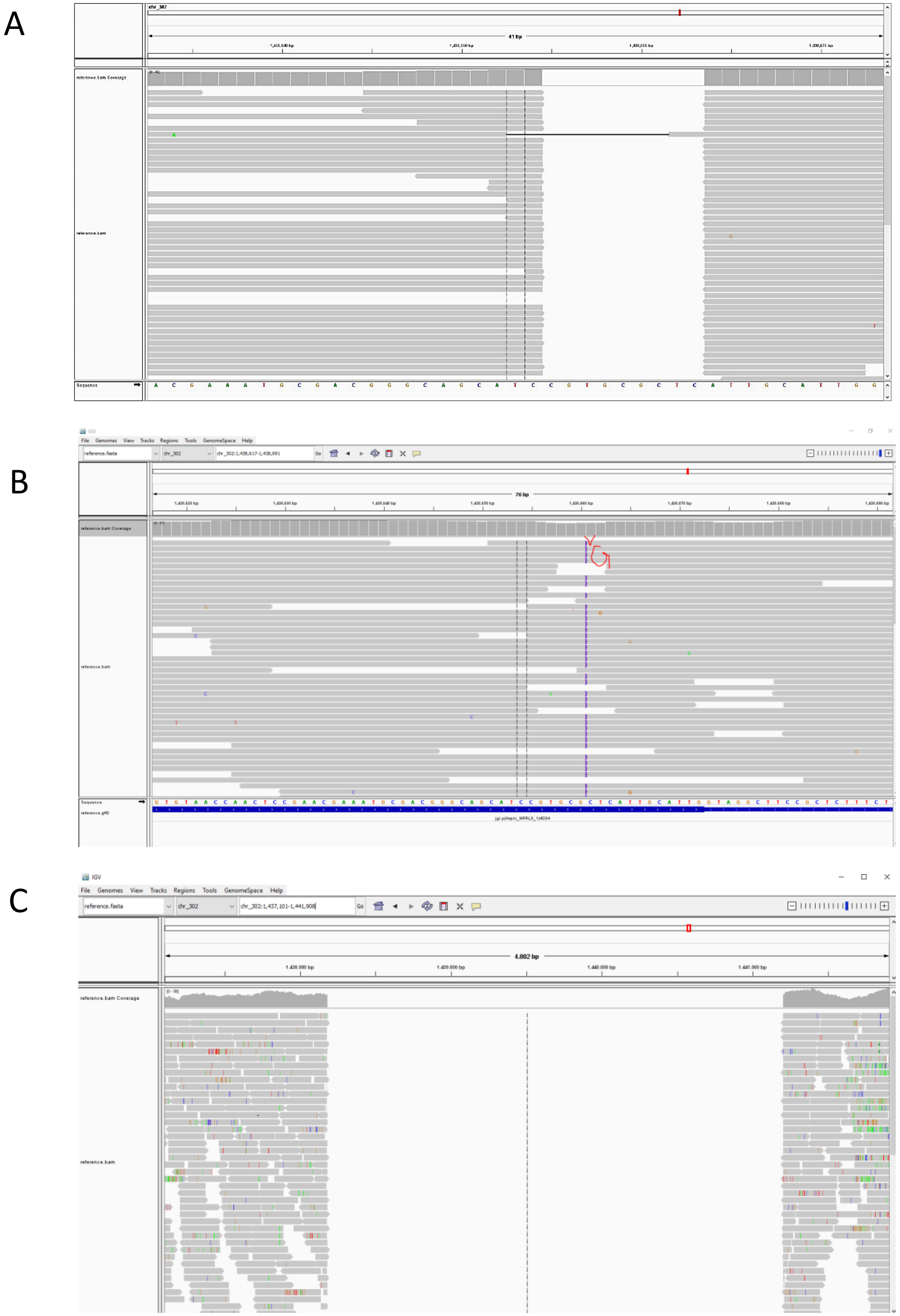

Supp. Fig. S2. WGS analysis confirmed different repairing patterns between HDR and NHEJ after CRISPR/Cas9 in *A. niger* wild type strains. (A) a 9bp deletion was detected in NHEJ repairing sample-13.10; (B) an insertion of extra G was detected in NHEJ repairing sample-13.35, which cause a frameshift mutation; (C) a full deletion of *XlnR* gene was detected on HDR repairing sample 13.26. All three detected variants occurred at CRISPR/Cas9 cutting position (chr_302: 1438653) resulted in dramatic function change of *XlnR* gene, which leads to a similar growth phenotype as showed in Fig. 1.

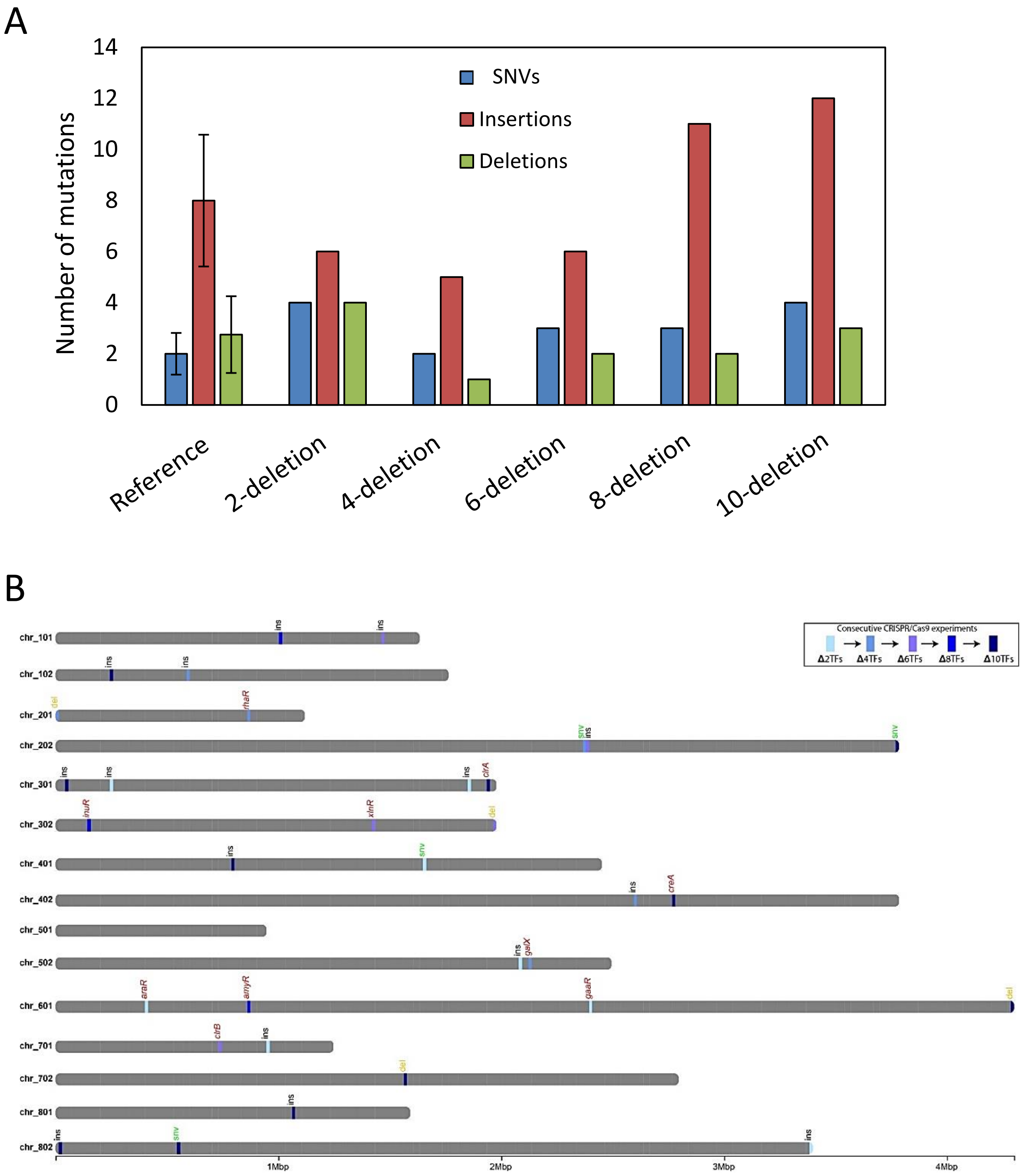

Supp. Fig S3. Occurrence of mutations upon consecutive CRISPR/Cas9 events.
Average number of SNVs, insertions and deletions in the Δ*kusA* background after consecutive CRISPR/Cas9 events. Reference: Δ*kusA* strain transformed with a no *cas9*-containing plasmid. 2-deletion*: Δ*araR*Δ*gaaR;* 4-deletion: Δ*araR*Δ*gaaR*Δ*rhaR*Δ*galX;* *6-deletion*: Δ*araR*Δ*gaaR*Δ*rhaR*Δ*galX*Δ*xlnR*Δ*clrB;*
8-deletion: Δ*araR*Δ*gaaR*Δ*rhaR*Δ*galX*Δ*xlnR*Δ*clrB*Δ*amyR*Δ*inuR;*
10-deletion: Δ*araR*Δ*gaaR*Δ*rhaR*Δ*galX*Δ*xlnR*Δ*clrB* Δ*amyR*Δ*inuR*Δ*clrA*Δ*creA.*
*Note that the 2-deletion strain is not the direct parental of the 4-deletion strain.
(B) Schematic representation of a minimal set of genetic variants observed during consecutive CRISPR/Cas9 gene editing in *A. niger* Δ*kusA* strains. As shown, mutations were randomly distributed accross the genome.

Suppl. Table S1. Average of mutations identified in the selected *A. niger* strains after WGS.
Data show mean ± Standard Deviation (SD) of the replicate samples. Number of replicates (n) are indicated in brackets

	Transformation control (no Cas9)		Transformation with Cas9, no gRNA		Transformation with Cas9 and gRNA cassette			
	wild type (n= 8)	Δ*kusA* (n= 4)	wild type (n= 11)	Δ*kusA* (n= 3)	Wild type (NHEJ) (n= 13)	Wild type (HR) (n= 13)	Wild type (NHEJ+HR) (n=26)	Δ*kusA* (n= 3)
**SNVs**	181 ± 78	2 ± 0.8	183 ± 89	7 ± 9	317 ± 159	282 ± 149	300 ±152	4 ± 1
**Insertions**	10 ± 3	8 ± 2	9 ± 1	8 ± 2	10 ± 4	10 ± 2	10 ±3	7 ± 2
**Deletions**	6 ± 2	2 ± 1	6 ± 3	2 ± 1	7 ± 3	6 ± 3	7 ±3	3 ± 2

Suppl. Table S2. Statistical analysis. Significance was referred to $p < 0.05$

Mutations	Comparisons	*p* value	Significance (*)
**SNVs**	wt no_cas9 VS. Δ*kusA* no_cas9	0.001	*
	wt no_cas9 VS. wt_cas9	0.973	
	Δ*kusA*_no_cas9 VS. Δ*kusA*_cas9	0.329	
	wt_Δ*xlnR*_NHEJ VS. wt_Δ*xlnR*_HR	0.569	
	wt_cas9 VS. Δ*kusA*_cas9	0.006	*
	wt_cas9 VS. wt_Δ*xlnR* (NHEJ+HR)	0.023	*
	Δ*kusA*_cas9 VS. Δ*kusA*_Δ*xlnR*	0.616	
	wt_Δ*xlnR* VS. Δ*kusA*_Δ*xlnR*	0.002	*
**Insertions**	wt no_cas9 VS. Δ*kusA* no_cas9	0.343	
	wt no_cas9 VS. wt_cas9	0.525	
	Δ*kusA*_no_cas9 VS. Δ*kusA*_cas9	0.867	
	wt_Δ*xlnR*_NHEJ VS. wt_Δ*xlnR*_HR	0.814	
	wt_cas9 VS. Δ*kusA*_cas9	0.528	
	wt_cas9 VS. wt_Δ*xlnR* (NHEJ+HR)	0.181	
	Δ*kusA*_cas9 VS. Δ*kusA*_Δ*xlnR*	0.741	
	wt_Δ*xlnR* VS. Δ*kusA*_Δ*xlnR*	0.139	
**Deletions**	wt no_cas9 VS. Δ*kusA* no_cas9	0.034	*
	wt no_cas9 VS. wt_cas9	0.935	
	Δ*kusA*_no_cas9 VS. Δ*kusA*_cas9	0.945	
	wt_Δ*xlnR*_NHEJ VS. wt_Δ*xlnR*_HR	0.446	
	wt_cas9 VS. Δ*kusA*_cas9	0.084	
	wt_cas9 VS. wt_Δ*xlnR* (NHEJ+HR)	0.444	
	Δ*kusA*_cas9 VS. Δ*kusA*_Δ*xlnR*	0.829	
	wt_Δ*xlnR* VS. Δ*kusA*_Δ*xlnR*	0.037	*

Suppl. Table S3. Primers used in this study

Primer ID	Use	TM (ºC)	Sequence (5' → 3')[a]	Purpose[b]	Reference
P1-gRNA	F	57.3	CAACCTCCAATCCAATTTGACTCCGCCGAA CGTACTG	gRNA	(38)
P2-gRNA	R	54.1	ACTACTCTACCACTATTTGAAAAGCAAAAA AGGAAGGTACAAAAAAGC	gRNA	(38)
Fw-screen	F	47.9	TTTTCTCTTCCATTTACGC	cse	(27)
Rev-screen	R	53.1	GGGGATCATAATAGTACTAGCCA	cse	(27)
P3-*xlnR*	R	53.2	**CGGTCTCCTGGCGAGTATGC**GACGAGCTT ACTCGTTTCG	gRNA	(23)
P4-*xlnR*	F	49.3	**GCATACTCGCCAGGAGACCG**GTTTTAGAG CTAGAAATAGCAAG	gRNA	(23)
An-*xlnR*_5F	F	55.4	GTGTGTGTGTGAGAGAGAAAGG	RT (outside), csa	(23)
An-*xlnR*_5R	R	55.1	GCATCTCATCATCAGCCGTGTGGAAAGTG AGGTATTCAGACCG	RT	(23)
An-*xlnR*_3F	F	57.6	CGGTCTGAATACCTCACTTTCCACACGGCT GATGATGAGATGC	RT	(23)
An-*xlnR*_3R	R	57.2	GACGAGAGGAGTTGGTAGCG	RT (outside), csa	(23)
An-*xlnR*-NEST_5F	F	55.7	CTTTCTCGTGGGTTCTTCACC	RT	(23)
An-*xlnR*-NEST_3R	R	56.6	GGATGTAGTCGTCCAGGAGG	RT	(23)
P3-*gaaR*	R	53.2	**CGTCGCGTCCATGCCGAACC**GACGAGCTT ACTCGTTTCG	gRNA	(56)
P4-*gaaR*	F	49.3	**GGTTCGGCATGGACGCGACG**GTTTTAGA GCTAGAAATAGCAAG	gRNA	(56)
An-*gaaR*_5F	F	56.5	AAAGCAATCACGGCAACTGG	RT (outside), csa	(56)
An-*gaaR*_5R	R	55.2	GGGTATCATCGCTCTAGTATTCACTGATGT GGGAAGCGAATAGAGG	RT	(56)
An-*gaaR*_3F	F	55.6	CCTCTATTCGCTTCCCACATCAGTGAATAC TAGAGCGATGATACCC	RT	(56)
An-*gaaR*_3R	R	56.1	TGTAGAATACTTGCCCATAGGTCG	RT (outside), csa	(56)
An-*gaaR*-NEST_5F	F	55.9	AGAATAGCTTCTCTTGCTAGACTGG	RT	(56)
An-*gaaR*-NEST_3R	R	56	CGTGGACGCATTCATCTGTATG	RT	(56)
P3-*araR*	R	53.2	**CCCAGAAAGTCAGGGCACAC**GACGAGCT TACTCGTTTCG	gRNA	(56)
P4-*araR*	F	49.3	**GTGTGCCCTGACTTTCTGGG**GTTTTAGAG CTAGAAATAGCAAG	gRNA	(56)
An-*araR*_5F	F	59.2	GTCCGCAAGTTGTGTGGTGG	RT (outside), csa	(56)
An-*araR*_5R	R	56.2	GCATCGGTGCTGTGAGAAACGGAATCGC AGTCTGATGAAACG	RT	(56)
An-*araR*_3F	F	56.7	CGTTTCATCAGACTGCGATTCCGTTTCTCA CAGCACCGATGC	RT	(56)
An-*araR*_3R	R	57.7	AACCGAGAAGCCCAGTTTCG	RT (outside), csa	(56)
An-*araR*-NEST_5F	F	57.8	GAAGCGACCTCATAGCGACC	RT	(56)
An-*araR*-NEST-3R	R	56.7	ATGCCAGAAACATGCGATGC	RT	(56)
P3-*rhaR*	R	53.2	**CGCTGTGATCCCGTTCCAGA**GACGAGCTT ACTCGTTTCG	gRNA	(56)
P4-*rhaR*	F	49.3	**TCTGGAACGGGATCACAGCG**GTTTTAGA GCTAGAAATAGCAAG	gRNA	(56)
An-*rhaR*_5F	F	56.5	GTGCGGATCTCTTTGATGCG	RT (outside), csa	(56)

An-*rhaR*_5R	R	56.1	GCGATAAACTGCCTCAAAGATGTTTTATC GGGAGATAGACAGGTGC	RT	(56)
An-*rhaR*_3F	F	55.7	GCACCTGTCTATCTCCCGATAAAACATCTT TGAGGCAGTTTATCGC	RT	(56)
An-*rhaR*_3R	R	55.4	CATCTTGACTTAGTGAACAGGAGC	RT (outside), csa	(56)
An-*rhaR*-NEST_5F	F	55	CAGTAGTTTAGCGGAGTGGG	RT	(56)
An-*rhaR*-NEST_3R	R	55.7	ACTCACCTCTTCTCTCATTCTCG	RT	(56)
P3-*galX*	R	53.2	**ACGCGTTTGGTGGACACTAT**GACGAGCTT ACTCGTTTCG	gRNA	(56)
P4-*galX*	F	49.3	**ATAGTGTCCACCAAACGCGT**GTTTTAGAG CTAGAAATAGCAAG	gRNA	(56)
An-*galX*_5F	F	56.4	CGTAGTTGAATGCGACCTGC	RT	(56)
An-*galX*_5R	R	56.7	CCTTTCAGAGCTTTGAGATGGAAGAGGGA AGAATGAGAAGTCAACCG	RT	(56)
An-*galX*_3F	F	55.7	CGGTTGACTTCTCATTCTTCCCTCTTCCATC TCAAAGCTCTGAAAGG	RT	(56)
An-*galX*_3R	R	56.9	AACAATTCAATTCGCAGAAGCAGC	RT (outside), csa	(56)
An-*galX*-NEST_5F	F	57.5	GCTCAAACTGCGGACAATGC	RT (outside), csa	(56)
An-*galX*-NEST_3R	R	56.4	CCAGTGCGATCAGAAACACG	RT	(56)
P3-*clrB*	R	53.2	**TCCTTTCTTCAGACCTGAGC**GACGAGCTTA CTCGTTTCG	gRNA	(24)
P4-*clrB*	F	49.3	**GCTCAGGTCTGAAGAAAGGA**GTTTTAGA GCTAGAAATAGCAAG	gRNA	(24)
An-*clrB*_5F/NEST_5F	F	56.7	ATCACACAACCCTTCTCGTACC	RT	(24)
An-*clrB*_5R	R	56.4	CGATAGCGAATCCTAGCAGTTGCTGGAGT TTTTGTTTGACGG	RT	(24)
An-*clrB*_3F	F	55.5	ACTGCTAGGATTCGCTATCGCTGTACTTAC AGGGTGCAGC	RT	(24)
An-*clrB*_3R/NEST_3R	R	55	GGATGGATCGTCTTAGGATGC	RT	(24)
An-*clrB*_F	F	57.5	GTGTGACTGTCCCCTTCTTCC	csa	(24)
An-*clrB*_R	R	56.4	TTCCTTATGTAGCCCCAGAGC	csa	(24)
P3-*amyR*	R	53.2	**TGCGATAAGTGTCGACGCCT**GACGAGCTT ACTCGTTTCG	gRNA	(Kun *et al*, unpublished)
P4-*amyR*	F	49.3	**AGGCGTCGACACTTATCGCA**GTTTTAGAG CTAGAAATAGCAAG	gRNA	(Kun *et al*, unpublished)
An-*amyR*_5F	F	57.1	TGGGATGTTCACCAGTGTTACG	RT (outside), csa	(Kun *et al*, unpublished)
An-*amyR*_5R	R	57.9	CGATAGCGAATCCTAGCAGTGCGGAGACA AGTGTGACTCC	RT	(Kun *et al*, unpublished)
An-*amyR*_3F	F	55.8	ACTGCTAGGATTCGCTATCGCAACTACGA CGATGACGATGC	RT	(Kun *et al*, unpublished)
An-*amyR*_3R	R	57.8	CACCGTGACCCAGAGAAAGG	RT (outside), csa	(Kun *et al*, unpublished)
An-*amyR*-NEST_5F	F	57.3	GAGCCTCAGACTCTGTCAGC	RT	(Kun *et al*, unpublished)
An-*amyR*-NEST_3R	R	55.4	TCCACCACCATCAAAATCACC	RT	(Kun *et al*, unpublished)
P3-*inuR*	R	53.2	**ACGTCTTCTTCTCGAGCTAA**GACGAGCTT ACTCGTTTCG	gRNA	(Kun *et al*, unpublished)
P4-*inuR*	F	49.3	**TTAGCTCGAGAAGAAGACGT**GTTTTAGA GCTAGAAATAGCAAG	gRNA	(Kun *et al*, unpublished)
An-*inuR*_5F	F	57	TCGGAGGAGATACGCAAAGC	RT (outside), csa	(Kun *et al*, unpublished)

An-*inuR*_5R	R	55.7	CGATAGCGAATCCTAGCAGTCTCACGAGT CATAAGCATTGTCG	RT	(Kun *et al*, unpublished)
An-*inuR*_3F	F	55.7	ACTGCTAGGATTCGCTATCGTGGCGTTCT GGTTTTATCACG	RT	(Kun *et al*, unpublished)
An-*inuR*_3R	R	57.7	ATTGACGGGCATGAGTGTCC	RT (outside), csa	(Kun *et al*, unpublished)
An-*inuR*-NEST_5F	F	57.4	TAAGAACAACGACCCCATCGC	RT	(Kun *et al*, unpublished)
An-*inuR*-NEST_3R	R	58.5	AAGGGAAACCGCCAATCAGC	RT	(Kun *et al*, unpublished)
P3-*clrA*	R	53.2	**TGGAAGAATTCGATGACAGG**GACGAGCT TACTCGTTTCG	gRNA	(24)
P4-*clrA*	F	49.3	**CCTGTCATCGAATTCTTCCA**GTTTTAGAGC TAGAAATAGCAAG	gRNA	(24)
An-*clrA*_5F/NEST_5F	F	55.1	TCAGCGAAACCAGTAAGAACG	RT	(24)
An-*clrA*_5R	R	54.9	CGATAGCGAATCCTAGCAGTGTTGTTGTG TTCAAGGGTGC	RT	(24)
An-*clrA*_3F	F	55.6	ACTGCTAGGATTCGCTATCGCATCATACGC ATCTTGTCAGTCC	RT	(24)
An-*clrA*_3R/NEST_3R	R	56.4	GGCAGTATTAACCAGGCTTGC	RT	(24)
An-*clrA*_F	F	56.9	TGTCAGTCTCGCCAACATACC	csa	(24)
An-*clrA*_R	R	55.8	AGTGTCAAGATGCTGTCTTTTTGG	csa	(24)
P3-*creA*	R	53.2	**CGGTGAGTGAGCGGGAGTCG**GACGAGCT TACTCGTTTCG	gRNA	(Kun *et al*, unpublished)
P4-*creA*	F	49.3	**CGACTCCCGCTCACTCACCG**GTTTTAGAGC TAGAAATAGCAAG	gRNA	(Kun *et al*, unpublished)
An-*creA*_5F	F	57	ACTTCTCCAGACCACACAGC	RT (outside), csa	(Kun *et al*, unpublished)
An-*creA*_5R	R	55	CGATAGCGAATCCTAGCAGTCGACGAGG GTAAAAAGGTAGG	RT	(Kun *et al*, unpublished)
An-*creA*_3F	F	56.6	ACTGCTAGGATTCGCTATCGCGAACATTCT TCAGCCACACG	RT	(Kun *et al*, unpublished)
An-*creA*_3R	R	55	TCATTCTGACTGACGCAATCC	RT (outside), csa	(Kun *et al*, unpublished)
An-*creA*-NEST_5F	F	56.6	TAGGCAACTGATGGAGACACC	RT	(Kun *et al*, unpublished)
An-*creA*-NEST_3R	R	56.9	GACGTTTGGGGTAAACAAAGCC	RT	(Kun *et al*, unpublished)

F: forward, R: reverse, TM: temperature of melting

[a]The gRNA sequence is highlighted in bold

[b]gRNA single guide, RNA RT repair template, BC with barcodes, TR traceability, cse colony screening for *E. coli*, csa colony screening for *A. niger*
